# Comparison of absorption and excretion of test compounds in sucking versus chewing pests

**DOI:** 10.1371/journal.pone.0321302

**Published:** 2025-04-28

**Authors:** Clara I. Römer, Roman Ashauer, Beate I. Escher, Juliane Hollender, René Burkhard, Kristin Höfer, Michel Muehlebach, Anke Buchholz

**Affiliations:** 1 Syngenta Crop Protection AG, Research Biology and Chemistry, Stein, Switzerland; 2 Syngenta Crop Protection AG, Basel, Switzerland,; 3 Department of Environment, University of York, Wentworth Way, Heslington, York, United Kingdom; 4 Department of Cell Toxicology, Helmholtz Centre for Environmental Research – UFZ, Leipzig, Germany; 5 Eberhard Karls University Tübingen, Environmental Toxicology, Tübingen, Germany; 6 Eawag, Swiss Federal Institute of Aquatic Science and Technology, Dübendorf, Switzerland; 7 Institute of Biogeochemistry and Pollution Dynamics, ETH Zürich, Zürich, Switzerland,; 8 Syngenta Crop Protection AG, Münchwilen, Switzerland; University of Naples Federico II: Universita degli Studi di Napoli Federico II, ITALY

## Abstract

A critical understanding of how pests interact with active ingredients is essential for the development of new insect control solutions to maintain crop quality and quantity by reducing insect damage. Absorption of insecticides into insect bodies of targeted pest species is the first critical step that confounds the efficacy of insecticides. This study investigated how different feeding behaviour of two pests, *Myzus persicae* and *Spodoptera littoralis*, affects the absorption, metabolism, and excretion (AME) of seven insecticidally inactive test compounds. A feeding contact assay for the chewing pest (Lepidopteran larvae) and an oral ingestion assay for the sucking pest (aphids) was used to investigate the AME of test compounds with agrochemical-like structural motifs. The standardized assays comprised of an exposure period with treated diet and a subsequent depuration period with untreated diet. The results showed that *S. littoralis* larvae differed from *M. persicae* in their compound quantities absorbed into the insect body and in their excretion products at the end of the exposure or depuration periods. We suggest that this is caused by their different ingestion types and rates resulting in different absorption and excretion quantities. Further, we found differences in the metabolism (timing and biotransformation pathways) of compounds between both species. Notably, certain compounds remained detectable in both pests after the depuration period, suggesting compound and species-specific metabolism and excretion. Our results highlight the complex interplay between feeding biology of insects, in particular the critical role of excretion products, and the exposure to different compounds that lead to species-specific AME.

## Highlights

- A new method described compound uptake and excretion in a sucking and chewing pest
- Chemical fate was described in an exposure (absorption) and depuration (elimination) period
- Feeding behaviour, biotransformation and compound properties influenced uptake and excretion
- Aphids had a high dietary intake to body mass ratio, but low total compound uptake into insect body
- Low lipophilic compounds had highest levels of compound quantities in excretion products

## 1. Introduction

Tailored insect control strategies are important to reduce the crop damage caused by various pests and to ensure yield quality and quantity. The use of insecticides remains a key component in this regard and innovative solutions in modern agricultural chemistry are continuously required [1–6].

However, their successful use is increasingly undermined by challenges such as the development of resistance to insecticides and pest shifts in crops [2,7–9]. It is imperative that the use of insect control solutions is carefully selected considering pest biology and crop growth cycles to secure crop protection [10,11]. Crop protection products get typically applied to crops by foliar spray. There are two principal application scenarios, i.e., curative or preventative treatments. In a curative spray, pests are directly exposed to the spray solution, whereas in a preventative treatment, pests get exposed to treated foliage after application [12].

The Absorption, Distribution, Metabolism, Excretion (ADME) framework supports the comprehensive understanding of the chemical fate of compounds within an organism. The effective dose on the leaf surface is determining the absorption of compounds by the insect as first step [13]. There are two dominant uptake routes into the insect, the oral and the contact absorption [14–16]. The life stage and feeding behaviour of the insect influence the oral absorption by active feeding and the contact absorption by passive diffusion across the insect cuticle. The internal distribution in insect tissues originates then from several permeation steps across different biomembranes. This subsequently determines the effective dose at internal target sites such as the nervous system or endocrine glands. The metabolism (or biotransformation) of parent compounds by enzymes or a sequestration into fat could detoxify the organism from these compounds [17,18]. Oxidative transformation increases the hydrophilicity of compounds and thereby facilitates their excretion. Whereas lipophilic compounds are more likely to accumulate in the fat body.

Phytophagous insects create different excretion products, frass, like feces (fecal pellets) or honeydew according to their diet. Excretion may include preceding metabolic processes. Further elimination of compounds (detoxification) strategies are enhanced excretion rates, regurgitation, or egg-laying [19,20]. Overall detoxification is directly related to the kinetics of compound elimination, including metabolic processes and excretion mechanism [21,22].

Crop damaging insects exhibit different feeding behaviours on various plant organs and tissues; each resulting in different damage types, but also different absorption routes of crop protection products [18,23]. Within this study we focus on two feeding types of foliar pests. *Myzus persicae* (Sulzer, 1776; Hemiptera: Aphididae) and *Spodoptera littoralis* (Boisduval, 1833; Lepidoptera: Noctuidae) are representative pests of great agronomic importance with wide distribution and often simplified as sucking and chewing pest [24,25].One prominent feeding type is represented by sucking and piercing insects. They possess specialised mouthparts such as stylets, which allow the ingestion of cell liquids and plant saps containing insecticides [26]. Hemipteran species, such as aphids, are so called sap feeders, i.e., their stylets take the sap from the plant vascular tissue (xylem and phloem) [25]. The resulting damage may be caused less by the direct ingestion of sap than by the transmission of viruses and other diseases [27].

While chewing and biting insects equipped with mandibles cut pieces of leaf material; notable examples include Lepidoptera larvae (caterpillars) [28]. This physical damage leads to reduced photosynthetic capacity, stunted growth and, in severe cases, to total pant collapse. In an agronomic context, this feeding damage can lead to significant reductions in crop yield and quality, up to and including total production loss. In order to develop effective insecticides against such diverse insect pests with the lowest possible environmental impact, it is important to understand the interaction of foliar applied compounds with the target pest. Optimal bioavailability of an insecticide in target pests facilitates favourable toxicokinetics. During lead optimization, to discover structurally novel compounds, researchers modify the central core structure (scaffold) of the active lead. This process alters affinities, selectivities, and ADME properties [29]. Such ‘scaffold hopping’ preserves the toxophore - the functional group interacting with the target protein - while changing the surrounding structure. Various approaches exist for characterizing pesticide-likeness [30], thereby guiding the design of new compounds with desired chemical fate. However, standardised assays for quantitative ADME comparisons across different pest species are rare, limiting the validation of these techniques.

In this context, we developed a *Spodoptera littoralis* bioassay in which larvae were exposed by feeding and contact with treated leaves. The assay design followed the established approach in toxicokinetics which comprises an exposure period followed by a depuration period. The depuration period was initiated by transferring the larvae to untreated leaves. The excretion processes in both periods, exposure, and depuration, reflect larva’s capability of compound elimination (detoxification) in addition to metabolism [31].

In a similar way, we present here the first time an exposure/ depuration bioassay for *Myzus persicae*. An artificial diet was prepared for the aphid (sachet) assay to provide different test compounds in an imbibable manner and at comparable exposure quantities. A leaf disk assay would carry the risk of compound specific foliar penetration rates and therefore unknown exposure quantities at the aphid feeding sites, the plant vascular tissues. This limits the exposure route to oral absorption in our assay. This should be acceptable as parthenogenetically reproducing aphids settle close to the initial female and nymphs and females spend most of their lifespan on a limited leaf area from which they are puncturing the leaf. Both bioassays allow the description and comparison of the chemical fate of test compounds in terms of absorption, metabolism, and excretion. Toxicological effects of insecticides could impair, e.g., insect’s movement, feeding activity, digestion and excretion. Therefore, insecticidally inactive test compounds with agrochemical scaffolds (i.e., with representative physicochemical properties but without toxophores) were selected to compare the unaffected A(D)ME between the chewing and sucking pest.

## 1. Materials and methods

### 1.1. Test compounds

**Fig. 1:**
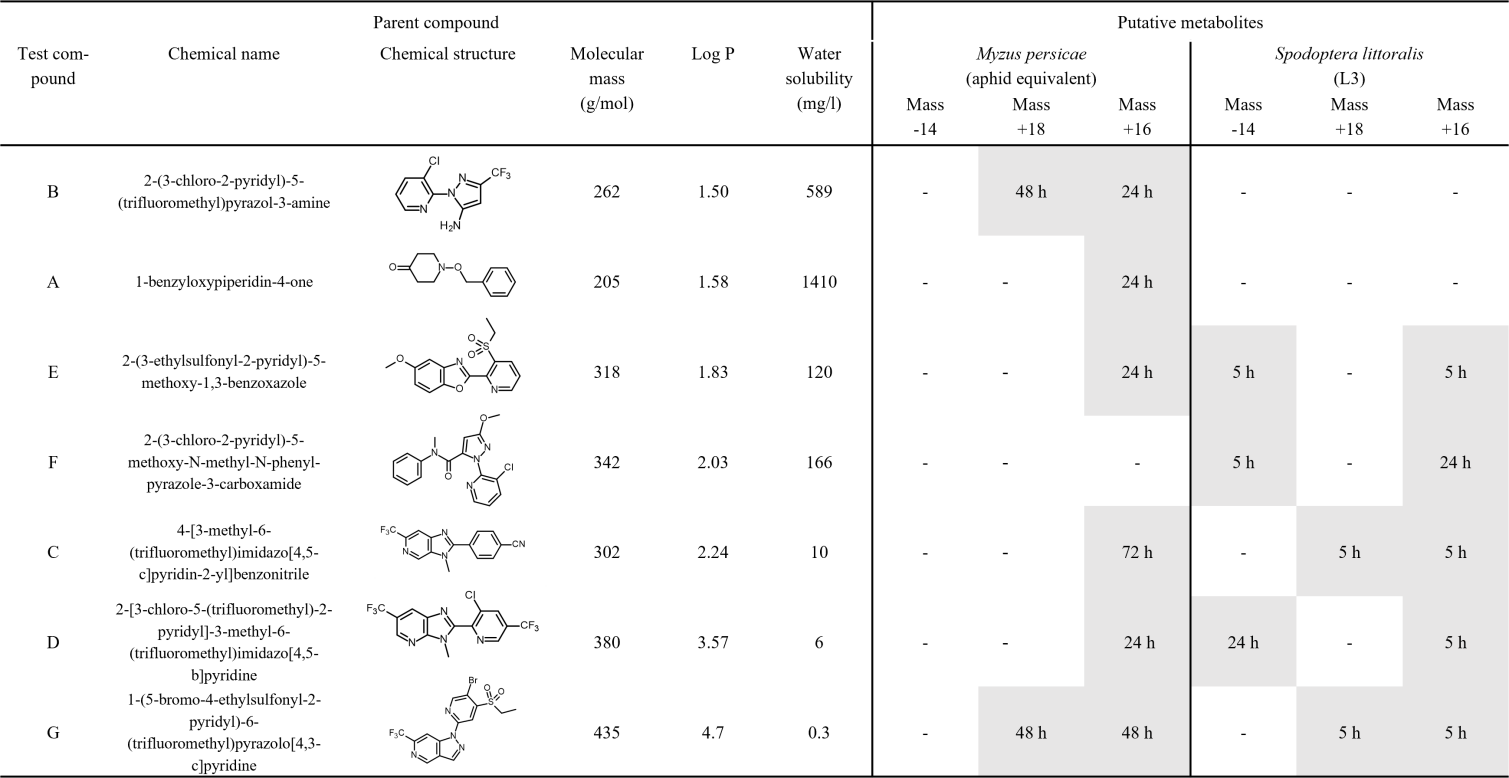
First time of detection of putative metabolites in insect samples of *Myzus persicae* (calculated equivalent based on aphid counts), and *Spodoptera littoralis* (larvae). Chemical name, chemical structure, molecular mass, measured log P, and water solubility of test compounds (A-G). Putative metabolites detected by mass difference (in Dalton) to given parent compound. All metabolites detected in the exposure period were also detected at all subsequent sampling time points (i.e., entire depuration period).

Seven insecticidally inactive test compounds with agrochemical related scaffolds were synthesised in house (≥ 95% purity). They ranged in lipophilicity, log P 1.50–4.7, and molecular mass, 146–433 g/mol (Fig. 1). The test compounds A to D were already described in a previous study building a toxicokinetic model based on this *S. littoralis* feeding-contact assay [31]. All compounds were dissolved at 2000 mg/L in water containing 15% acetonitrile (ACN) (gradient grade for analytics 99.9%) as solvent and pipetted onto leaf disks for larval assay. A high rate of 0.1 mg compound per leaf disk or diet was chosen to ensure good analytical detection of compounds in all biological matrices (insects and excreta). In addition, this rate did not show any adverse effects on larval performance parameters. Aqueous diet solutions contained 100 mg/L for aphid assay.

### 1.2. Oral ingestion assay– *Myzus persicae*

Many similar oral ingestion assays have been described since Mittler and Dadd [24]. In this study, aphids were feeding on an aqueous artificial diet mimicking phloem sap as their natural food source. This assay was designed to record compound quantities in aphids and their excreted honeydew over time. Aphids consumed treated artificial diet for 72 hours whereby they were exposed to test compounds. They were subsequently transferred to untreated food for a 72-hour depuration period. Aphids and honeydew were sampled during both periods of the experiment.

#### 1.2.1. Assay design and diet preparation.

**Fig 2:**
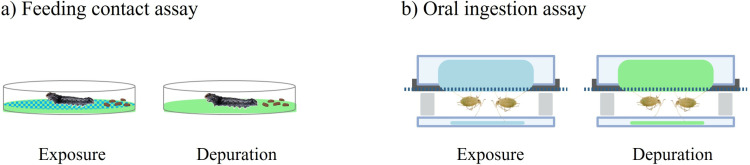
(a) Schematic overview of the *Spodoptera littoralis* feeding contact assay in a 12-well microtiter plate with either compound-treated or untreated leaf disk. (b) Schematic overview of the *Myzus persicae* oral ingestion assay in a 24-well microtiter plate, with either compound-treated or untreated diet. Blue = exposure preventative pipetted on leaf disk or in artificial diet, green = no treatment with compound. (Created with BioRender.com).

The aqueous artificial diet solution contained sucrose, minerals and amino acids according to [22]. For the exposure period, 1 mL of the artificial diet containing 0.1 mg of the dissolved test compound was added to 12 wells (replicates) of a 24-well microtiter plate (MT-plate, Falcon^TM^, Northfield, Minnesota, USA). Control treatments contained the artificial diet with the corresponding solvent quantity. The MT-plates were then covered with a layer of stretched Parafilm^TM^ and a perforated plate supporting aphid infestation on separate wells (Fig 2). For the depuration period, 24-well MT-plates were prepared in the same way, but the artificial diet did not contain test compounds.

#### 1.2.2. Infestation of aphids.

**Fig 3:**
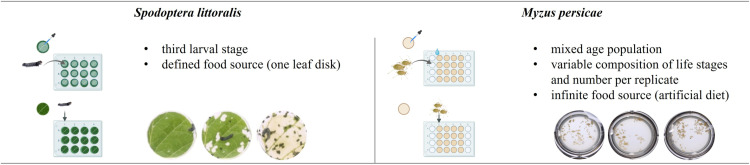
Bioassay conditions for *Spodoptera littoralis* and *Myzus persicae* highlighting the key differences. (Created with BioRender.com).

The Green peach aphids (*Myzus persicae*) originate from an asexual, wingless, laboratory strain that had not been exposed to insecticides before. Aphids were reared as mixed age population on pea seedlings under standardised conditions (20 ± 1 °C, 60 ± 10% RH, 16-hour light/8-hour dark cycle). Tips of infested pea seedlings were cut and placed into a Petri dish containing a dry filter paper and which was covered with a cotton filter (Ø 14.5 cm). Aphids of all life stages were allowed to migrate from the drying pea seedling onto the filter paper within two hours. Only these vital aphids were used for experimentation. Aphid populations of 15–30 individuals were brushed onto the individual wells of the MT plates (Figs 2 and 3). Aphids readily started feeding through the Parafilm™ membrane. A cardboard was then placed to close the infested MT plate upside down. After 15 minutes incubation the cardboard and any non–feeding aphids were removed. An empty MT plate was placed under the infested MT plate to collect the honeydew. Since mixed age populations were used adult females may produce nymphs so that the number of individuals per well (replicate) could increase during experimentation.

#### 1.2.3. Sampling.

Since the number of infested aphids varied between wells and over time, the MT plates were photographed prior to insect sampling to capture the number of individuals by counting. Aphid and diet samples were taken at 0, 24, 48, 72 (end of exposure), 75, 80 and 144 h (end of depuration). Honeydew was collected at the end of the exposure (72 h) and depuration (144 h) period by dissolving it from the plate with 1000 µL ACN. Here, samples were pooled from all replicates (12 wells) per treatment. All sample types were transferred to 2.5 mL tubes (MP Biomedicals™ FastPrep-24™ 5G, Lucerne Chem AG, Lucerne, Switzerland) and immediately frozen at -80 °C to stop any metabolism.

### 1.3. Feeding contact assay – *Spodoptera littoralis*

The feeding-contact assay with the Egyptian cotton leafworm, *Spodoptera. littoralis* larvae, was previously described in detail [31]. *S. littoralis* was reared in the laboratory under standardised conditions (23 ± 1 °C, 55% ± 10% RH), including an in-house artificial diet for both, adults and larvae. This laboratory strain has not been exposed to insecticides before. Single synchronised third instar larvae were exposed to applied test compounds (0.1 mg pipetted in 50 µL onto soybean leaf disks, Ø 20 mm) under standardised conditions (25 ± 1 °C, 55 ± 10% RH, 16-hour light/8-hour dark cycle). The assay included a 24-hour exposure period during which each individual larva was able to consume the entire leaf disk, followed by a transfer of each larva to an untreated leaf disk and a 24-hour depuration period. Both, oral ingestion and contact absorption, are considered as field-relevant uptake routes. Therefore, it is not possible to distinguish, for example, whether the compound(s) detected in larva bodies were absorbed internally or adsorbed on insect surfaces. Samples of larvae, leaf disks and fecal pellets were collected within one larval stage at various time-points (1 h, 5 h, 24 h, 25 h, 29 h and 48 h) for chemical analysis.

### 1.4. Compound quantification

The same chemical analysis method was applied to all biological matrices whereas the extraction methods differed for diet, insect, and excretion samples [31]. All samples were processed as the total mass of the given biological matrix. The wet weight of samples was measured using a Sartorius-balance (BCE124I-1S Entris® II, Data Weighing Systems, Inc., Wood Dale, IL, USA). All samples were homogenised using a macerator with a ceramic ball (Ø 6.35 mm) and dissolved in 500 µL ACN and extracted for 3 h. Afterwards samples were centrifuged at 9000 rpm for 2 min. Diet (leaf disk or artificial diet), fecal pellets and honeydew sample extracts were additionally filtered through a 0.20 µm pore size filter (CHROMAFIL®Xtra PET-20/13, Macherey-Nagel GmbH and Co.KG, Düren, Germany).

The quantity of test compounds in the biological matrices was determined by ultra-high performance liquid chromatography-mass spectrometry (UHPLC-MS) using ACN and water as solvents. Spectra of all samples for parent compounds and their metabolite residues were recorded on a Waters Corporation Mass Spectrometer (Xevo TQ-XS Triple Quadrupole Mass Spectrometer) equipped with an electrospray ionization (ESI+) source. Putative metabolites (identified as mass changes in Dalton) were scanned using negative ion mode (ESI-) in a mass range of 120–1000 Da. The quantification of putative metabolites was semi-quantitative because reference standards were not available and had to be calculated by comparing its detected quantities with the detected amounts of the parent compound within the given calibration series.

### 1.5. Compound quantities in insect bodies and excretion products

Total compound quantities were measured for individual replicates of insect samples. They were expressed either as compound quantity per individual *S. littoralis* larva or per ‘aphid equivalent’. The *M. persicae* samples were standardised by dividing the measured compound quantity by the counted number of aphids at the sampling time-point. This was relevant since replicates (wells) contained a variable number of individuals. However, this approach does not consider differences in age and size of aphid individuals and the time-course of their reproduction.

### 1.6. Comparison of compound quantities in insects and excretion products

At the end of the exposure and depuration period, the quantities of the parent compound measured in insect bodies and in excretion products (honeydew or fecal pellets) were compared. The honeydew samples represent the total quantity of each treatment group at the end of the exposure and depuration period. These compound quantities were correlated with the aphid number counted in all twelve replicates at the sampling time-point. This approach intends to provide a similar comparability to the compound quantities in aphid equivalents. Fecal pellets were sampled and measured for each replicate (*S. littoralis* larva) and time-point.

### 1.7. Calculation of diet uptake to body mass ratio

To further understand the relationship between compound quantities in insects and the ingestion rate of diet (diet uptake), we calculated the diet uptake (leaf disk or artificial diet) in relation to insect body masses. This relationship is defined as ‘diet uptake to body mass ratio’.


$$\text{diet }\;\;\;\;\;\text{ uptake }\;\;\;\;\;\text{ to }\;\;\;\;\;\text{ body }\;\;\;\;\;\text{ mass }\;\;\;\;\;\text{ ratio}{\text{end}}_{exposure}=\text{diet }\;\;\;\;\text{ uptake }\;\;\;\;/\;\;\;\;\text{ insect }\;\;\;\;\text{ wet }\;\;\;\;\text{ weight }\;\;\;\;$$ (1)

We could not quantify the ingestion rate of *M. persicae* in our assay. Therefore, as an approximation, we used the ingestion rate of 0.022 µL h^-1^ as reported by Rhodes *et al*. [32] for pea aphids (*Acyrthosiphon pisum*) feeding on an artificial diet. We calculated the diet uptake to body mass ratio by dividing the postulated ingestion volume after 72 h (1.58 µL) by the average wet weight of an aphid equivalent (Ø 0.42 mg).

For *S. littoralis* it was determined at the end of the exposure period (24 h) when the larvae had consumed the entire leaf disk (average weight Ø 23 mg). The average weight of a *S. littoralis* larvae (Ø 17.14 mg) at 24 h was used in the calculation.

## 2. Results

### 2.1. Measured compound quantities in both pest species

**Fig 4:**
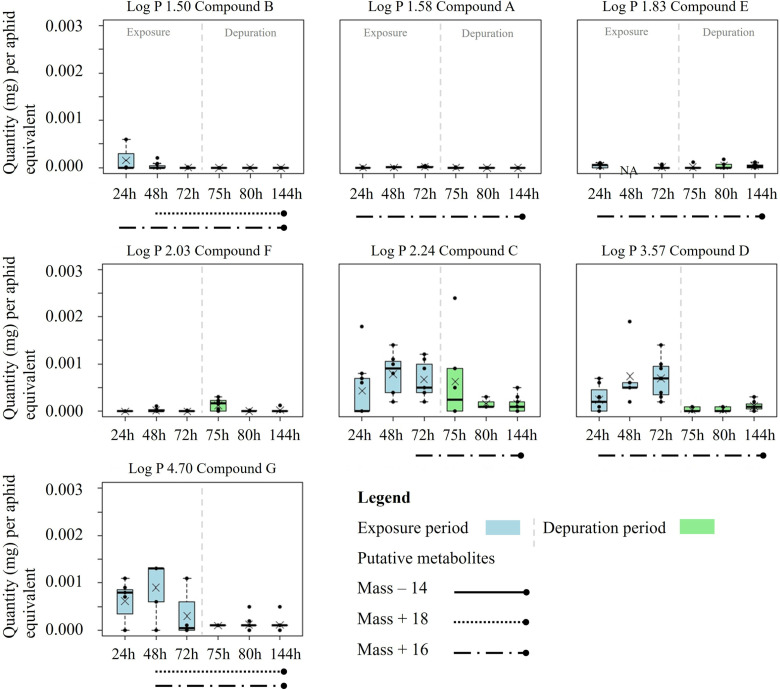
Compound quantities (mg) per *Myzus persicae* aphid (calculated equivalent based on aphid counts) bodies over time. The compounds are arranged in order of increasing log P (Fig. 1). Aphids fed on treated artificial diet for 72 h (exposure), followed by a 72-h feeding period on untreated diet (depuration) (n=8 replicates; aphid population per sampling time). Occurrence of putative metabolites (mass changes, Fig. 1) over time represented as different line types below the graphs, respectively. Boxplots show interquartile ranges, raw data points, mean (X**)**, and medians (black lines). Whiskers not exceeding 1.5 × of the interquartile range extend to the maximum and minimum. Outliers are shown as circles. Figures created using R (version 3.5.3; R Core Team, 2020).

**Fig 5:**
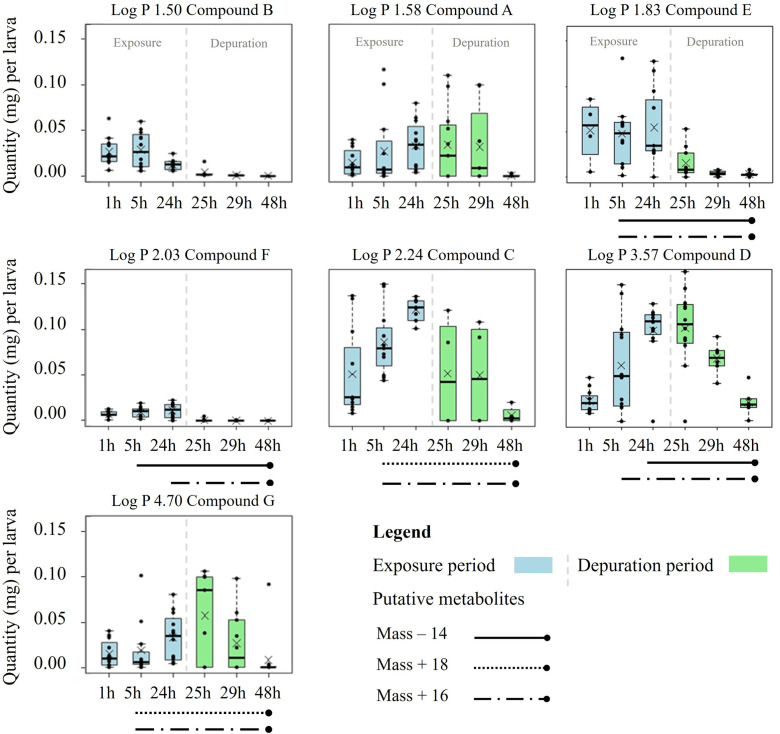
Compound quantities (mg) per *Spodoptera littoralis* larval bodies over time. The compounds are arranged in order of increasing log P (Fig. 1). Larvae consumed one treated leaf disk within 24 h (exposure), followed by one untreated leaf disk in the subsequent 24 h (depuration), (n=12 larvae). Occurrence of putative metabolites (mass changes, Fig. 1) over time represented as different line types below the graphs, respectively. Boxplots show interquartile ranges, raw data points, mean (X), and medians (black lines). Whiskers not exceeding 1.5 × of the interquartile range extend to the maximum and minimum. Outliers are shown as circles. Data on parent compound quantities for 4 test compounds (A-D) were taken from [31]. Figures created using R (version 3.5.3; R Core Team, 2020).

Notable differences in parent compound quantities were measured in aphid (*M. persicae)* and entire larval (*S. littoralis*) bodies in exposure and depuration periods of respective bioassays (Figs 4 and 5).

No uniform time courses of parent compound quantities were observed in *M. persicae* aphids (calculated equivalent based on aphid counts) (Fig 4). Compounds C and D showed an increase in aphid bodies during the exposure to treated diet followed by a decrease during the depuration. In contrast, the measured quantities of parent compound G decreased already during the exposure period with the maximum at 48 h. Compounds A, E, and F demonstrated comparable parent compound quantities in aphids during both periods. Compound D had the highest total quantity (0.021 mg per aphid equivalent) in *M. persicae* aphids. Conversely, compounds A, B, and E had the lowest total quantity in aphids. At the end of the depuration period (144 h), compounds C, D, E, and G were still detectable in aphids.

All compound quantities in *S. littoralis* larvae, except for compound B, increased during the exposure and decreased during the depuration period (Fig 5). Each compound reached its maximum quantity at different sampling time points during the exposure or depuration. Compound F reached its maximum quantity after 1 h of larval exposure on treated leaf disks, followed by compound B after 5 h. Compounds A, C, D, and E showed the time point of their maximum quantity after 24 h at the end of the exposure period. In contrast, compound G reached its maximum quantity after 25 h, which represents the first hour of the depuration period. Compound C had the highest (0.085 mg per larva) and compound F the lowest (0.029 mg per larva) overall quantity in larval bodies. Only compounds C and D remained above the limit of detection in larval bodies after 48 h at the end of the depuration period.

### 2.2. Parent compound quantities in excretion relative to insect bodies

**Fig 6:**
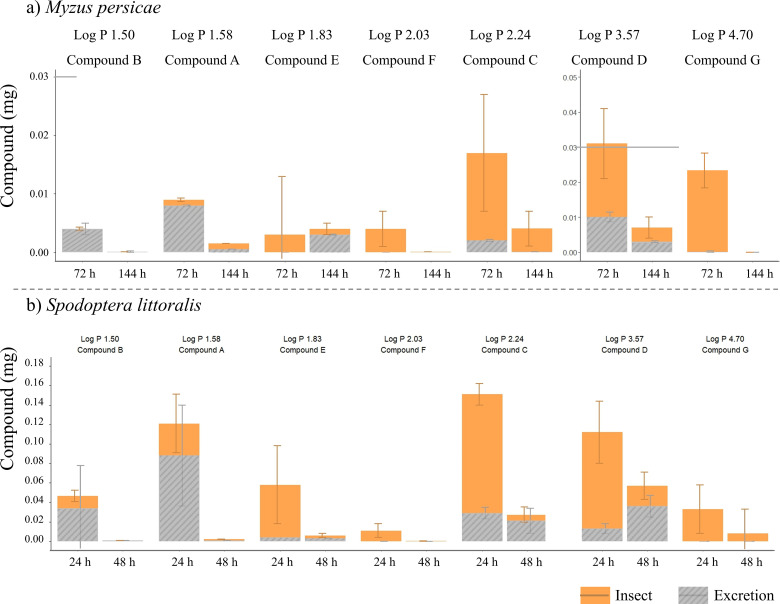
Distribution of measured parent compounds in insects (bodies) and excretion products (fecal pellets or honeydew). (**a)** Compound quantity (mg) per *Myzus persicae* aphid (calculated equivalent based on aphid counts, n=8) at the end of the exposure (0-72 h) and depuration (72-144 h) period, respectively. Aphids fed on treated artificial diet for 72 h, followed by a 72-h feeding period on untreated diet. (**b)** Compound quantity (mg) per *Spodoptera littoralis* larva (n=12) at the end of the exposure (0-24 h) and depuration (24-48 h) period, respectively. Larvae consumed one treated leaf disk within 24 h, followed by one untreated leaf disk in the subsequent 24 h. Data on parent compound quantities for 4 test compounds (A-D) were taken from [31]. Stacked graphs show the mean (orange: in insect, hatched grey: in excretion) with standard error (grey lines). (Horizonal grey line shows the different y-axis scale for compound D). Note the different y-axis for *S. littoralis* and *M. persicae.* Figures created using R (version 3.5.3; R Core Team, 2020).

Compounds quantities in insect bodies of *M. persicae* and *S. littoralis* and their excretion products (honeydew and fecal pellets) differed between exposure and depuration periods (Fig 6). In both species no lethal effects of the compounds were observed.

#### a) *Myzus persicae.*

At the end of the exposure period (72 hours), compounds E, F, C, and D exhibited higher quantities in insects compared to their excretion product, whereas compounds B and A showed relative higher amounts in the honeydew (Fig 6a). Compound D displayed the highest quantity in the insect (aphid equivalent) at the end of the exposure period, followed by compounds G and C. In contrast, compound E exhibited the overall highest amount in honeydew followed by compound D at the end of the exposure period.

At the end of the depuration period (144 hours), relative higher quantities were found for compounds A, C, and D in insects, while relative higher quantities were measured for compound E in honeydew.

#### b) *Spodoptera littoralis.*

At the end of the exposure period (24 h), compounds C- G exhibited higher quantities in insects compared to their excreted fecal pellets (Fig. 6b). Conversely, compounds A and B showed higher quantities in fecal pellets than in insect bodies. Compound C reached the overall highest quantity in insects whereas compound A showed the highest quantity of parent compound in fecal pellets.

At the end of the depuration period (48 h), compounds E and G still displayed higher quantities in insects compared to their excretion products; while compounds C and F showed relative higher quantities in fecal pellets (Fig 6a).

### 2.3. Putative metabolism (biotransformation)

The putative metabolites observed in *Myzus persicae* and *Spodoptera littoralis* are described as mass changes (-14, +18, +16 Da) of test compounds over time (Fig. 1). For *Myzus persicae* no mass decrease but mass increase by +18 and +16 Da were observed over time. After 48 h of aphid exposure to treated diet, metabolites with a mass change of + 18 Da were observed for compounds B and G. A mass change of + 16 Da was observed for compounds A, B, D and E after 24 h, for compound G after 48 h, and for compound C after 72 h exposure.

In *Spodoptera littoralis* (Fig. 1), all three putative pathways were observed (mass changes of -14, +18, +16 Da). All metabolites were found at shorter retention times in the reverse phase chromatography compared to the parent compounds indicating an increase of the polarity. A metabolite with a mass decrease of -14 Da, a potential demethylation, was detected for compounds E and F after 5 h larval exposure to treated leaf disks and for compound D after 24 h exposure. Another metabolite with a mass increase of +18 Da, a potential water addition, was observed for compounds C and G after 5 h exposure. The mass increase of +16 Da (oxidation) was detected for all compounds at 5 h (compounds C, D, E, G) and 24 h exposure (compound F). No metabolites were observed for compounds A and B.

## 3. Discussion

This study investigated two agronomically relevant pests, the green peach aphid, *M. persicae*, and the Egyptian cotton leafworm, *S. littoralis*, in dedicated bioassays consisting of exposure to insecticidally inactive compounds followed by a depuration period. The results on measured parent compound quantities and putative metabolites suggest that the absorption (uptake), metabolism (biotransformation), and excretion (elimination) are influenced by the different feeding biology and/or physiology of both pest species.

Both bioassays differed regarding the absorption routes during the exposure period (Fig 2). In the *M. persicae* assay oral absorption dominated because aphids were feeding on an artificial diet mimicking plant sap. The precise internal distribution of test compounds in different insect tissues remains unknown, as only entire insect bodies were analysed. The *S. littoralis* bioassay made of larvae feeding on treated leaf disks combined active oral absorption (larvae cutting leaf pieces with their mandibles) and passive contact (larvae crawling on spray deposits on leaf surfaces). This combination of two principal absorption routes is typical for field conditions where pests get preventatively exposed to foliar applied crop protection products.

### Absorption (uptake) and excretion of compounds

Both, absorption, and excretion mechanisms, in species such as *M. persicae* and *S. littoralis* are influenced by insect physiology, feeding biology, and the chemical properties of ingested compounds. Differences in the excretion (elimination) of compounds may be due to variations in A(D)ME processes within the insect body. These processes have an impact on the residence time of compounds within the insect, which in turn affects the quantity of compound found in excretion products. Typically, compounds with shorter residence time are excreted more rapidly and thus may undergo no or less extensive metabolic transformation. *M. persicae* and *S. littoralis* showed different patterns of compound quantities in their bodies and excretion products, honeydew, or fecal pellets (Fig 6).

In honeydew of *M. persicae* the measured quantities of compound D were notably higher at the end of the exposure period than at the end of the depuration period; whereas the opposite was detected for compound E (Fig 6a). This suggests that compound D could already be excreted during its exposure whereas compound E may have had a longer residence time and could get gradually excreted during the subsequent depuration period.

Compounds with log P values lower than 1.58, explicitly A and B, were predominantly excreted already at the end of the exposure period for both species. In contrast, compounds with relative higher log P values (C: 2.24 and D: 3.57) were detected in fecal pellets of *S. littoralis* larvae in high quantities at the end of the depuration period (Fig 6b). These results are consistent with other *S. littoralis* studies, where consumed compounds remained unaltered at excretion [33].

Interestingly, compounds F and G were, regardless of the time of sampling, not detected in the excretion products of both pest species investigated (Fig 6). This observation strongly indicates that both compounds were completely metabolised and were therefore not excreted as parent compounds but as metabolites in honeydew or fecal pellets. This suggests biotransformation as an effective elimination pathway [21]. The predominance of parent compounds in excretion products may indicate either limited absorption potential in insect bodies and/ or a fast excretion process.

### Metabolism (biotransformation) of compounds

Metabolic processes have a crucial influence on the detectable quantities of parent compounds in given insect species, as they determine the concentration in the insect body, as well as in excretion products (honeydew or fecal pellets). In our study, we observed differences in the metabolism of test compounds, leading to different changes in their mass (Da). Specifically, we detected three prominent mass changes.

Metabolism within insects influences the dynamics and quantities of measured compounds. In aphids, the absorption of compound B seemed to decrease once a second metabolite appeared (Figs 1 and 4). Compounds A and E were continuously metabolised with the start of the exposure and no increase in parent compounds in aphid bodies was detected. The onset of metabolism of compounds C and G appears to be well correlated with the decrease of parent compounds. This suggests a faster biotransformation than the oral ingestion of compounds. However, the absorption of compound D into aphid bodies continued to increase throughout the exposure period, regardless of an early onset of metabolism.

*S. littoralis* indicated different biotransformation processes for compounds C, D, E, F and G (Figs 1 and 5). Notably, metabolites of compound E emerged after 5 hours, coinciding with a temporary drop in the parent compound quantity before it increased again with prolonged exposure. This suggests continuous absorption in parallel to biotransformation. During the exposure period compounds C, D and G demonstrated consistent increase in parent compounds in *S. littoralis* larvae irrespective to continuous metabolism. Conversely, the quantity of compound F did not increase once metabolic processes were detected, suggesting a higher biotransformation than uptake rate. The mass reduction of -14 Da was unique to *S. littoralis* larvae, suggesting a species-specific metabolic process, possibly mass change indicating demethylation, which could result in a more hydrophilic compound derivate which is easier to excrete (Fig. 1). Rup et al. [34] also described demethylation as biotransformation pathway for mustard aphid (*Lipaphis erysimi*) feeding on radish plants. The fact that we did not detect this pathway may be due to the different diet and sampling dates in our assays or it may actually indicate a true species difference. Metabolites of compounds A and B were not detected in *S. littoralis* larvae, but only in *M. persicae* (Fig. 1). Mass increases of +16 Da and +18 Da, indicative for oxidation processes such as hydroxylation and water addition, were observed in both species but not necessarily for each compound (Fig. 1). It is important to note that our results can only be taken as approximation due to possible limitations in our analytical method. Future research should aim for a comprehensive identification of metabolic pathways [35].

Our findings emphasize the complex interactions between the dynamics in compound absorption and metabolic processes across insect species and indicate that biotransformation alone is not always an adequate indicator of effective compound elimination. A thorough analysis and identification of metabolites would be essential to confirm these observations and to better understand the dynamics between absorption and biotransformation rates defining the resulting compound quantities in insect bodies (Figs 1 and 5).

### Significance of feeding biology

To better understand the physiological differences between both pest species investigated, the total diet uptake over the exposure period (larvae: 24 h; aphids 72 h) was calculated in relation to the respective body mass (see 2.7 in Material and Methods section). The resulting diet uptake to body mass ratio is 3.7 and 1.3 for *M. persicae* aphid equivalents and *S. littoralis* larvae, respectively. This reveals that aphids exhibit in general a 2.8 times higher absorption rate than *S. littoralis* larvae. In addition to the diet quantity, both insect species differ also significantly regarding their feeding style and therefore diet quality.

The diet of aphids feeding on the vascular tissue is typically rich in carbohydrates, but often deficient in essential amino acids (unfavourable carbon-nitrogen ratio) which affects ingestion rates and population dynamics [36].To compensate the nutritional limitations of their diet, *M. persicae* have evolved symbiotic relationships with gut bacteria that aid in extraction or synthesis of essential nutrients, particularly amino acids [37]. Their gut system is also adapted to this unique diet, with a neutral or slightly acidic foregut, an acidic midgut optimised for nutrient absorption and enzymatic activity, and a neutral or slightly acidic hindgut [38–40].

Feeding on foliar tissues provides a complex mixture of nutrients including carbohydrates, proteins and fats, as well as secondary plant metabolites such as alkaloids, terpenes, phenols, polyphenols or glycosides [40]. These compounds could act as chemical defence mechanism by, e.g., disrupting the digestion of herbivores [41]. Widespread polyphagous pests like *Spodoptera* have developed superfamilies of detoxification genes to arm themselves against plant toxins and xenobiotics [42]. Their intestinal pH varies from neutral to slightly acidic in the foregut and to very alkaline in the midgut. In the hindgut, the pH might drop to the level of the foregut [43,44]. The peritrophic matrix protects the epithelial cells of the midgut in Lepidoptera; a barrier which is absent in aphids [44,45]. Such fundamental differences in diet quantity, dietary composition, pH milieu and specialised symbiotic relationships define species specific differences in biotransformation pathways. Species differences occur already between members of the same family: Two aphid species feeding on rape cultivars demonstrated different strategies in handling the glucosinolate (GSL) content in their diet. *Myzus persicae* excreted GSL in honeydew whereas *Brevicoryne brassicae* accumulated GSL in the body [46]. Accordingly, the chemical fate and resulting uptake into internal insect tissues (bioavailability) will differ between pest species.

Another aspect is the residence time of the diet in the respective gut system. The 2.8 times higher diet uptake to body mass ratio for aphids also implies a faster gut passage which is expected to affect both, uptake into internal insect tissues and exposure to metabolic processes [36]. This might contribute to the overall low compound quantities measured in aphid bodies, the less diverse biotransformation compared to *S. littoralis* and the tendency for higher absorption rates with more lipophilic test compounds. However, comprehensive metabolite identifications (qualitative and quantitative) are required to substantiate such correlations.

Critical insights on these complex interdependencies are of high value to pesticide research. Good knowledge on factors affecting ADME in target and non-target pests could support chemistry design of new insect control solutions providing effective pest control with minimal environmental impact.

### Physicochemistry of test compounds

Poor bioavailability of pesticides in target organisms is one of the major bottlenecks in the discovery of pesticides. The molecular physicochemical characteristics can explain pesticide-likeness, including hydrophobicity, lipophilicity, electronic and structural properties, water solubility, and crystal packing [47]. Lipophilicity, most commonly referred to as the log P, is a key property in transport processes, including intestinal absorption, membrane permeability, protein binding, and distribution to different tissues and organs [48]. An increase in the molecular mass or size is often observed to be associated with lower solubility and poor penetration through membranes [49]. The McGowan volume is another molecular property known to influence absorption and distribution of agrochemicals [50].

**Fig 7:**
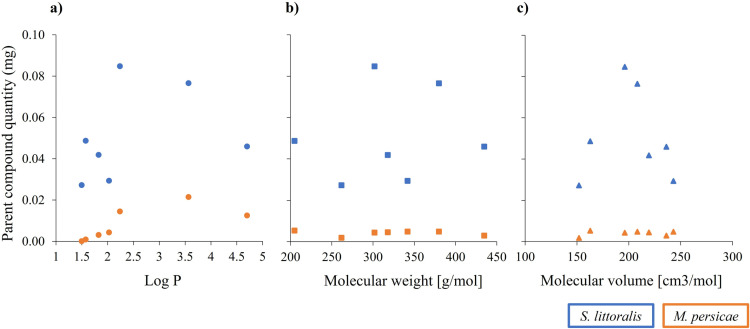
Parent compound quantities (mg) in *Spodoptera littoralis* (larvae, n=12) and *Myzus persicae* (calculated aphid equivalent, n=8) plotted against log P (a), molecular weight (b), and molecular volume (c) (Fig. 1). Dots represent the average of parent quantities (mg per insect) at the end of the exposure period. Data on parent compound quantities for 4 test compounds (A-D) were taken from [31].

One could take measured compound quantities in insect bodies as a reference on bioavailability for a given test compound (Fig. 7). Our results appear to indicate that compounds between a log P value of 2.2 (C) and 3.6 (D) demonstrated relative higher uptake into insect bodies of both species. The plot against molecular mass did not reveal an obvious correlation whereas the molecular volume suggests favourable absorption into *S. littoralis* larvae at a size of ca. 200 cm^3^ mol^-1^. Multi-variate data analyses on larger datasets will be required to better understand such complex interdependencies on bioavailability.

## Conclusions

This study highlighted the complex interactions involved in the uptake and elimination of different test compounds into two insect pests. Factors such as feeding behaviour, which determines diet quality and ingestion rates, the biotransformation capabilities of the insect and physicochemical properties of compounds, such as log P and molar volume, are all important. Aphids have a relative higher diet uptake to body mass ratio than *S. littoralis* larvae, but overall absorption quantities of compounds in aphid bodies remained lower.

Measured compound quantities in insect bodies and excretion products at the end of the exposure and depuration periods in our bioassays could give an indication on the residence time of compounds within insect bodies. Less lipophilic compounds were detected at higher quantities in insect excretion products (honeydew and fecal pellets) than in respective insect bodies. Whereas increasing lipophilicity of compounds resulted in relative higher quantities in insect bodies.

This highlights that the description of the chemical fate of compounds in insects requires the consideration of the entire ADME processes and dynamics. A snapshot on, e.g., concentrations in insects could cause misleading conclusions if excreted quantities get omitted. For example, unlike *S. littoralis,* we did not detect the mass change (in Da) indicating demethylation pathway in *M. persicae* in our sampling setup. Furthermore, differences in species specific metabolism (dynamics and biotransformation pathways) emphasize the need for a comprehensive analysis (i.e., metabolite identification and quantification). Follow-up research is required to substantiate the possibility of a species selectivity. Better understanding of species-specific differences in ADME, specifically in biotransformation, could pave the way for more selective pest control solutions by raising the effectiveness against target pests and minimising the impact on non-target organisms.

## Supporting Information

S1 file The Supporting Information provides the raw data of compound quantities in leaf disks, fecal pellets, larval bodies from the *Spodoptera* assay; and honeydew and aphid bodies from the *Myzus* assay. (PDF)
