# Supplementary material for: Comparison of absorption and excretion of test compounds in sucking versus chewing pests
Source: PLoS One. 2025 Apr 28;20(4):e0321302. doi: 10.1371/journal.pone.0321302 (PMC12036848; doi:10.1371/journal.pone.0321302)
Supplement: S1 file — (PDF) [file pone.0321302.s001.pdf]

Supporting Information

Comparison of absorption and excretion of test compounds in sucking versus chewing pests.

Clara I. Römer, Roman Ashauer, Beate I. Escher, Juliane Hollender Renè Burkhard, Kristin Höfer,
Michel Muehlebach Anke Buchholz

Data *Spodoptera littoralis*

Time is always in hours expressed in columns replicates are in rows.

Compound A – Leaf disk toxicokinetic bioassay

time	mg per leaf disk			
0	0.1	0.1	0.1	0.1
1	0.1	0.1	0.101	0.098
5	0.101	0.1	0.1	0.1
24	0.1	0.1	0.098	0.1
25	0.0000	0.0000	0.0000	0.0000
29	0.0000	0.0000	0.0000	0.0000
48	0.0000	0.0000	0.0000	0.0000

Compound B – Leaf disk toxicokinetic bioassay

time	mg per leaf disk			
0	0.1	0.1	0.098	0.1
1	0.07	0.065	0.062	0.1
5	0.05	0.065	0.068	0.056
24	0.05	0.065	0.068	0.05
25	0.0000	0.0000	0.0000	0.0000
29	0.0000	0.0000	0.0000	0.0000
48	0.0000	0.0000	0.0000	0.0000

Compound C– Leaf disk toxicokinetic bioassay

time	mg per leaf disk			
0	0.098	0.101	0.098	0.1
1	0.1	0.1	0.1	0.098
5	0.101	0.101	0.101	0.1
24	0.1	0.089	0.1	0.1
25	0.0000	0.0000	0.0000	0.0000
29	0.0000	0.0000	0.0000	0.0000
48	0.0000	0.0000	0.0000	0.0000

Compound D– Leaf disk toxicokinetic bioassay

time	mg per leaf disk			
0	0.1	0.098	0.101	0.1
1	0.098	0.1	0.1	0.1
5	0.098	0.1	0.09	0.1
24	0.098	0.101	0.09	0.1
25	0.0000	0.0000	0.0000	0.0000
29	0.0000	0.0000	0.0000	0.0000
48	0.0000	0.0000	0.0000	0.0000

Compound E – Leaf disk toxicokinetic bioassay

time	mg per leaf disk			
0	0.1	0.1	0.1	0.1
1	0.1	0.1	0.101	0.098
5	0.1	0.1	0.1	0.1
24	0.1	0.1	0.103	0.098
25	0.0000	0.0000	0.0000	0.0000
29	0.0000	0.0000	0.0000	0.0000
48	0.0000	0.0000	0.0000	0.0000

Compound F – Leaf disk toxicokinetic bioassay

time	mg per leaf disk			
0	0.1	0.1	0.1	0.101
1	0.1	0.1	0.1	0.1
5	0.1	0.1	0.101	0.098
24	0.1	0.1	0.1	0.099
25	0.0000	0.0000	0.0000	0.0000
29	0.0000	0.0000	0.0000	0.0000
48	0.0000	0.0000	0.0000	0.0000

Compound G – Leaf disk toxicokinetic bioassay

time	mg/mg leaf			
0	0.098	0.1	0.1	0.1
1	0.1	0.1	0.1	0.1
5	0.1	0.101	0.098	0.081
24	0.1	0.1	0.1	NaN
25	0.0000	0.0000	0.0000	0.0000
29	0.0000	0.0000	0.0000	0.0000
48	0.0000	0.0000	0.0000	0.0000

Data *Spodoptera littoralis* feces– Compound quantities

Time is always in hours.

Compound A – Leaf disk toxicokinetic bioassay: Feces

	mg per feces total	
	time	
replicate	0-24 h	0-48 h
1	0.125	0
2	0.125	0
3	0.104	0
4	0.104	0
5	0.146	0.001
6	0.146	0.001
7	0.063	0.001
8	0.063	0.001
9	0.000	0.001
10	0.000	0.001
11	NA	NA
12	NA	NA

Compound B – Leaf disk toxicokinetic bioassay: Feces

	mg per feces total	
	time	
replicate	0-24 h	0-48 h
1	0.021	0.001
2	0.021	0.001
3	0.000	0.000
4	0.000	0.000
5	0.000	0.000
6	0.021	0.001
7	0.021	0.001
8	0.021	0.001
9	0.125	0.001
10	0.125	NaN
11	0.021	NaN
12	NaN	NaN

Compound C– Leaf disk toxicokinetic bioassay: Feces

	mg per feces total	
	time	
replicate	0-24 h	0-48 h
1	0.036	0.031
2	0.036	0.031
3	0.023	0.020
4	0.023	0.000
5	NaN	NaN
6	NaN	NaN
7	NaN	NaN
8	NaN	NaN
9	NaN	NaN
10	NaN	NaN
11	NaN	NaN
12	NaN	NaN

Compound D – Leaf disk toxicokinetic bioassay: Feces

	mg per feces total	
	time	
replicate	0-24 h	0-48 h
1	0.019	0.024
2	0.019	0.024
3	0.010	0.053
4	0.010	0.054
5	0.006	0.041
6	NaN	0.041
7	NaN	0.023
8	NaN	0.022
9	NaN	0.035
10	NaN	0.033
11	NaN	0.043
12	NaN	0.044

Compound E– Leaf disk toxicokinetic bioassay: Feces

	mg per feces total	
	time	
replicate	0-24 h	0-48 h
1	0.004	0
2	0.002	0
3	0.006	0
4	0.001	0.001
5	0.004	0.001
6	NA	NA
7	NA	NA
8	NA	NA
9	NA	NA
10	NA	NA
11	NA	NA
12	NA	NA

Compound F – Leaf disk toxicokinetic bioassay: Feces

	mg per feces total	
	time	
replicate	0-24 h	0-48 h
1	NaN	NaN
2	NaN	NaN
3	NaN	NaN
4	NaN	NaN
5	NaN	NaN
6	NaN	NaN
7	NaN	NaN
8	NaN	NaN
9	NaN	NaN
10	NaN	NaN
11	NaN	NaN
12	NaN	NaN

Compound G– Leaf disk toxicokinetic bioassay: Feces

	mg per feces total	
	time	
replicate	0-24 h	0-48 h
1	NaN	NaN
2	NaN	NaN
3	NaN	NaN
4	NaN	NaN
5	NaN	NaN
6	NaN	NaN
7	NaN	NaN
8	NaN	NaN
9	NaN	NaN
10	NaN	NaN
11	NaN	NaN
12	NaN	NaN

Data *Spodoptera littoralis* – Compound quantities

Compound A – Leaf disk toxicokinetic bioassay: total body

time (h)	mg per larva											
0	0	0	0	0	0	0	0	0	0	0	0	0
1	0.036	0.013	0.022	0.033	0.012	0.002	0.007	0.001	0.003	0.007	0.04	0.001
5	0.006	0.009	0.002	0.051	0.101	0.004	0.008	0	0.117	0.004	0	0.025
24	0.006	0.04	0.061	0.064	0.005	0.01	0.047	0.004	0.08	0.03	0.012	0.038
25	0	0.11	0	0	0.035	0	0	0.022	0.052	0.06	NaN	0.098
29	0	0.0085	0.1	0.0085	0.099	0	0	0.038	NaN	NaN	NaN	NaN
48	0.001	0.001	0.003	0	0	0	0.001	0.001	0	0	NaN	0

Compound B – Leaf disk toxicokinetic bioassay: total body

time	mg per larva											
0	0	0	0	0	0	0	0	0	0	0	0	0
1	0.0156	0.0336	0.0205	0.02	0.0361	0.0169	0.0059	0.0152	0.023	0.0233	0.0633	0.0413
5	0.0344	0.0138	0.0481	0.0106	0.0059	0.0513	0.0097	0.0601	0.0057	0.0185	0.0425	0.0417
24	0.0151	NaN	0.0161	0.0146	0.0067	0.0169	0.0104	0.0051	NaN	0.0057	0.0107	0.0247
25	0.0014	0.0012	0.0019	0.002	0.001	0.0159	NaN	NaN	NaN	NaN	NaN	NaN
29	0.0008	0.0007	0.0011	0.0008	0.0006	0.0005	NaN	NaN	NaN	NaN	NaN	NaN
48	0.0002	0.0001	0	0	0	0	NaN	NaN	0.0002	0.0002	0.0001	NaN

Compound C – Leaf disk toxicokinetic bioassay: total body

time	mg per larva										
0	0	0	0	0	0	0	0	0	0	0	0
1	0.024	0.1369	0.1337	0.0124	0.0257	0.026	0.0156	0.0087	0.02	0.0631	0.0981
5	0.0933	0.0447	0.0502	0.1479	0.0473	0.0829	0.15	0.0729	0.1101	0.07	0.0796
24	0.1325	0.1367	0.1242	0.1172	0.1104	0.132	0.1103	0.1014	0.1311	NaN	NaN
25	0	0	0.086	0.1213	NaN	NaN	NaN	NaN	NaN	NaN	NaN
29	0.00001	0.1084	0.00001	0.0919	NaN	NaN	NaN	NaN	NaN	NaN	NaN
48	0.0049	0.0202	0	0	NaN	NaN	NaN	NaN	NaN	NaN	NaN

Compound D – Leaf disk toxicokinetic bioassay: total body

time (h)	mg per larva											
0	0	0	0	0	0	0	0	0	0	0	0	0
1	0.014	0.048	0.02	0.009	0.025	0.039	0.014	0.025	0.01	0.031	0.012	NaN
5	0.019	0	0.015	0.101	0.051	0.048	0.15	0.024	0	0.092	0.095	0.14
24	0.099	0	0.119	0.117	0.114	0.102	0.109	0.088	0.091	0.117	0.11	0.129
25	0.146	0	0.13	0.091	0.061	0.102	0.126	0.124	0.083	0.164	0.111	0.089
29	0.061	0.093	0.075	0.065	0.042	0.078	NaN	NaN	NaN	NaN	NaN	NaN
48	0.019	0.018	0.025	0.001	0.015	0.048	NaN	NaN	NaN	NaN	NaN	NaN

Compound E – Leaf disk toxicokinetic bioassay: total body

time	mg per larva											
0	0.0000	0.0000	0.0000	0.0000	0.0000	0.0000	0.0000	0.0000	0.0000	0.0000	0.0000	0.0000
1	0.0450	0.0060	0.0690	0.0860	NaN	NaN	NaN	NaN	NaN	NaN		NaN
5	0.0110	0.0590	0.0150	0.0560	0.0400	0.0020	0.0670	0.0600	NaN	0.1310	0.0380	NaN
24	0.0950	0.0270	0.0290	0.1170	0.0300	0.0000	0.0350	0.0760	0.1280	0.0300	0.0350	NaN
25	0.0090	0.0050	0.0070	0.0270	0.0000	0.0340	0.0530	0.0080	0.0110	NaN	0.0000	NaN
29	0.0030	0.0030	0.0020	0.0000	0.0070	0.0060	0.0050	0.003	0.0080	NaN	0.0040	NaN
48	0.0040	0.0030	0.0080	0.0030	0.0080	0.0030	0.0020	0.0030	0.0030	0.0030	0.0000	NaN

Compound F – Leaf disk toxicokinetic bioassay: total body

time	mg per larva											
0	0.0000	0.0000	0.0000	0.0000	0.0000	0.0000	0.0000	0.0000	0.0000	0.0000	0.0000	0.0000
1	NaN	NaN	0.0111	0.0103	0.0060	0.0049	0.0067	0.0131	NaN	0.0058	0.0013	0.0076
5	0.0106	0.0031	0.0196	0.0129	0.0159	0.0040	0.0022	NaN	NaN	0.0063	0.0105	NaN
24	0.0061	0.0037	0.0082	0.0015	0.0167	0.0163	0.0183	0.0231	0.0006	NaN	0.0197	NaN
25	0.0053	0.0009	0.0004	0.0002	0.0003	0.0003	NaN	0.0005	NaN	0.0002	0.0005	NaN
29	0.0002	0.0014	0.0002	NaN	0.0002	NaN	0.0001	0.0000	0.0002	0.0002	NaN	0.0002
48	0.0001	0.0002	0.0003	0.0000	0.0001	NaN	0.0001	0.0003	NaN	0.0002	0.0003	0.0001

Compound G – Leaf disk toxicokinetic bioassay: total body

time (h)	mg per larva											
0	0.0000	0.0000	0.0000	0.0000	0.0000	0.0000	0.0000	0.0000	0.0000	0.0000	0.0000	0.0000
1	0.0357	0.0132	0.0218	0.0332	0.0120	0.0024	0.0073	0.0011	0.0025	0.0068	0.0401	0.0000
5	0.0058	0.0086	0.0019	0.0505	0.1008	0.0043	0.0084	0.0000	0.0255	0.0041	0.0000	NaN
24	0.0058	0.0404	0.0605	0.0642	0.0046	0.0100	0.0470	0.0043	0.0800	0.0303	0.0121	0.0380
25	0.0000	NaN	0.0853	0.1001	NaN	0.0848	0.0994	0.0000	0.0000	0.0375	0.1057	NaN
29	0.0000	NaN	0.0000	0.0000	0.0346	0.0000	0.0000	0.0216	0.0518	0.0600	NaN	0.0981
48	0.0008	0.0010	0.0033	0.0000	0.0002	0.0000	0.0009	0.0011	0.0000	0.0000	0.0912	0.0000

Data *Myzus persicae* honeydew– Compound quantities

Time is always in hours.

Compound A – Artificial diet toxicokinetic bioassay: honeydew

mg per honeydew			
	time		
replicate	0-48 h	0-72 h	72-144 h
	0.004	0.013	0
	0.004	0.013	0
per well	0.0005	0.00163	0
	0.0005	0.00163	0

Compound B – Artificial diet toxicokinetic bioassay: honeydew

mg per honeydew			
	time		
replicate	0-48 h	0-72 h	72-144 h
	0.004	0.001	
	0.003		
per well	0.0005	0.00013	0
	0.00038	0	0

Compound C – Artificial diet toxicokinetic bioassay: honeydew

mg per honeydew			
	time		
replicate	0-48 h	0-72 h	72-144 h
	0.015		0.0003
	0.012		0.00028
per well	0.00188	0	3.7E-05
	0.0015	0	3.5E-05

Compound D – Artificial diet toxicokinetic bioassay: honeydew

mg per honeydew		
	time	
replicate	0-72 h	72-144 h
	0.076	0.001
	0.074	0.001
	0.099	0.004
	0.099	0.004
per well	0.0095	0.00013
	0.00925	0.00013
	0.01238	0.0005

Compound E – Artificial diet toxicokinetic bioassay: honeydew

mg per honeydew		
	time	
replicate	0-72 h	72-144 h
		0.025
		0.001
per well	0	0.00313
	0	0.00013

Data *Myzus persicae*– Compound quantities

Compound A – Artificial diet toxicokinetic bioassay: total body

time (h)	mg per aphids equivalent											
24	0.0002	NaN	NaN	0.0001	0.0001	0.0001	0.0000	0.0000	0.0000	0.0001	0.0001	0.0000
48	NaN	0.0003	0.0005	0.0003	NaN	0.0001	NaN	NaN	NaN	NaN	NaN	0.0003
72	0.0010	NaN	0.0006	0.0007	0.0011	0.0010	0.0010	0.0007	0.0014	0.0005	0.0006	0.0007
75	0.0001	0.0002	0.0003	0.0005	0.0001	0.0000	0.0000	0.0000	NaN	NaN	NaN	NaN
80	0.0000	0.0000	0.0000	0.0000	0.0000	NaN	NaN	NaN	NaN	NaN	NaN	NaN
144	0.0000	0.0001	0.0001	0.0000	0.0000	0.0000	0.0000	0.0000	NaN	NaN	NaN	NaN

Compound B – Artificial diet toxicokinetic bioassay: total body

time (h)	mg per aphids equivalent								
24	0.0001	NaN	0.0000	0.0006	NaN	NaN	NaN	NaN	NaN
48	0.0000	0.0010	0.0020	0.0000	0.0000	0.0000	0.0000	0.0000	0.0000
72	0.0000	0.0001	0.0000	0.0000	0.0000	0.0000	0.0000	0.0000	NaN
75	0.0000	0.0000	0.0000	0.0000	0.0000	0.0000	0.0000	0.0000	NaN
80	0.0000	0.0000	0.0000	0.0000	NaN	NaN	NaN	NaN	NaN
144	0.0000	0.0000	0.0000	0.0000	0.0000	0.0000	0.0000	NaN	NaN

Compound C – Artificial diet toxicokinetic bioassay: total body

time (h)	mg per aphids equivalent								
24	0.0143	0	0	0	0.0167	0	0.0129	0.0406	0
48	0.0316	0.0037	0.0086	0.0098	0.0209	0.0238	0.0214	0.0181	NaN
72	0.0266	0.0094	0.0102	0.0245	0.0088	0.0036	0.0187	NaN	NaN
75	NaN	NaN	0.0103	0	0.0204	0.0538	0	0	NaN
80	0.0071	0.003	NaN	NaN	NaN	0.0032	NaN	NaN	NaN
144	0.0038	0.0024	0.0031	0	0.0049	0.0107	0	0	0.0074

Compound D – Artificial diet toxicokinetic bioassay: total body

time (h)						mg per aphids equivalent				
24	0.0090	0.0000	0.0080	0.0000	0.0000	0.0000	0.0030	0.0000	0.0200	0.0170
48	0.0160	0.0560	0.0140	0.0190	0.0070	NaN	NaN	NaN	NaN	NaN
72	0.0430	0.0130	0.0270	0.0210	0.0290	0.0100	0.0070	NaN	NaN	NaN
75	0.0010	0.0020	0.0020	0.0010	0.0000	0.0000	NaN	NaN	NaN	NaN
80	0.1300	0.0000	0.0000	0.0020	0.0000	0.0020	NaN	NaN	NaN	NaN
144	0.0010	0.0030	0.0000	0.0100	0.0040	0.0050	0.0040	NaN	NaN	NaN

Compound E – Artificial diet toxicokinetic bioassay: total body

time	mg per aphids equivalent									
24	0.0010	0.0020	0.0010	0.0000	0.0020	0.0010	0.0010	0.0000	0.0000	NaN
48	NaN	NaN	NaN	NaN	NaN	NaN	NaN	NaN	NaN	0.1310
72	0.0000	0.0010	0.0020	0.0000	0.0000	0.0000	0.0000	0.0000	0.0000	0.0300
75	0.0030	0.0000	0.0000	0.0000	0.0000	0.0000	0.0000	0.0000	NaN	NaN
80	0.0020	0.0050	0.0000	0.0000	0.0020	0.0000	NaN	NaN	NaN	NaN
144	0.0030	0.0000	0.0000	0.0000	0.0020	0.0010	0.000	NaN	0.0010	0.0030

Compound F – Artificial diet toxicokinetic bioassay: total body

time	mg per aphids equivalent											
24	0.0010	0.0000	0.0002	0.0002	0.0011	0.0033	0.0000	0.0012	0.0000	0.0013	0.0000	0.0000
48	0.0000	0.0000	0.0000	0.0001	0.0000	0.0000	0.0000	0.0000	0.0000	0.0000	0.0007	0.0000
72	0.0000	0.0000	0.0069	0.0057	0.0089	0.0046	0.0066	0.0068	0.0000	0.0014	0.0057	0.0000
75	0.0006	0.0000	0.0004	0.0003	0.0005	0.0004	0.0000	0.0003	NaN	NaN	NaN	0.0003
80	0.0038	0.0000	NaN	0.0003	0.0001	0.0003	0.0003	NaN	NaN	NaN	NaN	NaN
144	0.0002	0.0000	0.0000	0.0000	0.0000	0.0000	0.0000	0.0007	NaN	NaN	NaN	NaN

Compound G – Artificial diet toxicokinetic bioassay: total body

time (h)	mg per aphids equivalent											
24	0.0139	0.0000	0.0000	0.0194	0.0141	0.0121	0.0157	NaN	NaN	NaN	NaN	NaN
48	0.0230	0.0233	0.0235	0.0007	NaN	0.0103	0.1200	NaN	NaN	NaN	NaN	NaN
72	0.0204	0.0010	0.0000	NaN	NaN	0.0000	NaN	NaN	NaN	NaN	NaN	NaN
75	0.0026	0.0024	NaN	NaN	NaN	NaN	NaN	NaN	NaN	NaN	NaN	NaN
80	0.0029	0.0005	0.0012	0.0010	0.0011	0.0022	0.0009	0.0092	0.0019	0.0017	0.0011	0.0003
144	0.0005	0.0012	0.0010	0.0011	0.0022	0.0009	0.0092	0.0019	0.0017	0.0011	0.0003	NaN
